# Joint Mobile Data Collection and Wireless Energy Transfer in Wireless Rechargeable Sensor Networks

**DOI:** 10.3390/s17081881

**Published:** 2017-08-16

**Authors:** Ping Zhong, Ya-Ting Li, Wei-Rong Liu, Gui-Hua Duan, Ying-Wen Chen, Neal Xiong

**Affiliations:** 1 School of Information Science and Engineering, Central South University, Changsha 410083, China; ping.zhong@csu.edu.cn (P.Z.); 703038@csu.edu.cn (Y.-T.L.); duangh@csu.edu.cn (G.-H.D.); xiongnaixue@gmail.com (N.X.); 2 College of Computer, National University of Defense Technology, Changsha 410073, China; csywchen@gmail.com; 3 Department of Mathematics and Computer Science, Northeastern State University, Tahlequah, OK 74464, USA

**Keywords:** data collection, wireless charging, network partition, adaptive anchor selection algorithm, optimization function

## Abstract

In wireless rechargeable sensor networks (WRSNs), there is a way to use mobile vehicles to charge node and collect data. It is a rational pattern to use two types of vehicles, one is for energy charging, and the other is for data collecting. These two types of vehicles, data collection vehicles (DCVs) and wireless charging vehicles (WCVs), are employed to achieve high efficiency in both data gathering and energy consumption. To handle the complex scheduling problem of multiple vehicles in large-scale networks, a twice-partition algorithm based on center points is proposed to divide the network into several parts. In addition, an anchor selection algorithm based on the tradeoff between neighbor amount and residual energy, named AS-NAE, is proposed to collect the zonal data. It can reduce the data transmission delay and the energy consumption for DCVs’ movement in the zonal. Besides, we design an optimization function to achieve maximum data throughput by adjusting data rate and link rate of each node. Finally, the effectiveness of proposed algorithm is validated by numerical simulation results in WRSNs.

## 1. Introduction

Wireless sensor networks are composed of a large number of sensor nodes distributed randomly in a particular area, which can be used for sensing data, information transmission, event monitoring and so on. However, most nodes in the network are powered by batteries and can only provide limited lifetime. Once the node is depleted of energy, it immediately enters a state of death. The battery of dead node needs to be replaced to reduce network channel cost. Now, solar energy, wind energy, tidal energy and other sustainable energy can be used to charge sensors continuously, the network is referred to as wireless rechargeable sensor network (WRSN) [1]. Although the method of gathering energy from the environment can solve the problem of node energy restriction to some extent, there are still many deficiencies, for example, insufficient energy density and instability. Recently, wireless energy transfer (WET) technology [2] has provided a new approach for solving sensors energy problem. Instead of obtaining energy from the environment, WET uses wireless transmitting devices to charge the nodes sending charging requests. The technology is more controllable and stable in energy density and energy sources. Nowadays, WET technology is widely used in various networks, for example, Radio Frequency Identification Networks (RFIDs), Wireless Powered Communication Networks (WPCNs) [3], Simultaneous wireless information and power transfer (SWIPT) [4].

The realization of WET is divided into the Near-field Coupling and the Far-field Radio Frequency Power Transfer. Near-field Coupling is divided into inductive coupling, resonance coupling and capacitive coupling. The Far-field Radio Frequency Power Transfer includes Directive RF Power Beamforming and Non-directive RF Power Transfer [5]. In this paper, we use wireless charging technology based on resonance coupling, which can transfer large amounts of energy in an efficient way [6,7]. There are four kinds of scenarios used for wireless charging, point provisioning, path provisioning, multihop provisioning and landmark provisioning [8]. Point provisioning employs static charger to supply energy for static nodes, and path provisioning uses static charger to supply energy for dynamic nodes. Multihop provisioning uses a static charger responsible for all nodes, since the sensor nodes equipped with energy transfer function can transfer their energy to neighbor nodes. By deploying a series of landmarks in advance, a dynamic charger is applied to charge nodes along the fixed path landmarks in Landmark provisioning [5]. The charging scenario in this paper is similar to the landmark provisioning and the dynamic charger is replaced by Wireless Charging Vehicles (WCVs) equipped with a resonant coil. In addition, WCVs can cover the whole network and move to the charging requested nodes for wireless charging [9]. The energy charging scheme is charging on demand and the requested nodes are arranged based on certain rules to maximize the nodes lifetime.

In addition to collect energy from the external environment, saving energy consumption is also an effective method to extend network lifetime, for example, constructing appropriate data transmission and collection protocol to save energy overhead in data collection process [10,11,12]. Liu et al. [13] proposed an effective clustering algorithm based on classical routing algorithm to mitigate the hotspot problem and collect more data packets. In [14], a static sink node collected data from each node with a multihop mode in the network. This method would cause energy problem called hotspot. Due to a large number of data packets transmitted and received, the nodes around the sink node would consume their own energy faster. In addition, it might cause node death, service interruption, packet loss and other problems. Zhao et al. [15] used a vehicle to complete data collection and wireless charging tasks in the network. A single vehicle eased the energy issue to some certain extent. However, the sojourn time of each node was limited. Charging and data collection could not be carried out at the same time. Besides, the charging time could not be ignored, which would result in higher data gathering delay, lower data amount and poorer network performance. Wang et al. [16] used a single data collection vehicle (DCV) and multiple wireless charging vehicles to complete data collection and energy transmission respectively in the network. Many charging vehicles needed to be arranged the proper charging path and charging queue. Otherwise, the vehicle energy consumption would be increasing and the nodes could not be charged efficiently.

**Figure 1:**
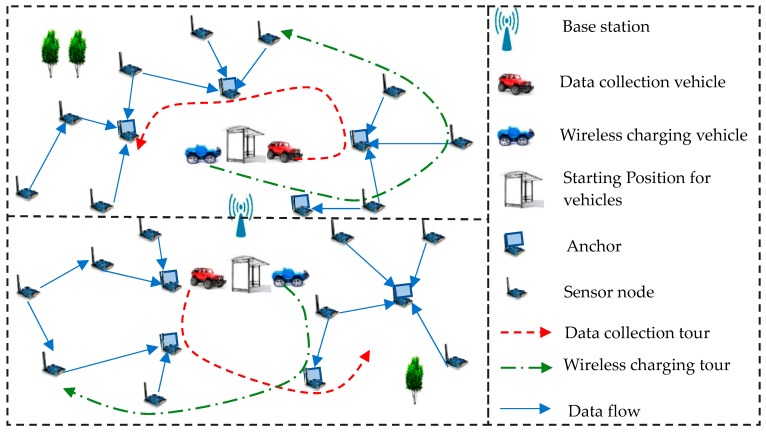
Network model and components.

Accordingly, we jointly consider mobile data collection and wireless charging in a large-scale network. Figure 1 illustrates the scenario of the whole network. The sensing field is divided into two divisions. The base station is located at the center of the area, which is used for processing data and charging for vehicles. Each division has a WCV and a DCV touring its field to charge nodes and collect data respectively. The characteristics of network data collection delay depends only on DCV, link rate and data generate rate. This avoids the collection delay due to charging time [15]. To reduce energy consumption, a subset of nodes is selected as anchor. Other nodes need to transmit its data to anchor by multi-hops. However, a series of problems need to be solved in this situation. First, is how to divide the large-scale network properly based on the limited vehicles. Second, is how to select the starting point of DCV and WCV in sub-regions. This can reduce the travelling distance of the vehicles and the energy consumption of data transmission. Third is which nodes should be chosen as anchors to collect more data in each area. To answer the above questions, we will propose a set of algorithms to solve these problems.

The main contributions of our work are summarized as follows. First, we propose a twice-partition algorithm based on center points for large-scale networks, which can simplify the schedule of vehicles in a more comprehensive way. Then, based on the distance and routing hops, we calculate the appropriate vehicles’ starting points to reduce the energy consumption on vehicles movement and forwarding data. Third, an anchor selection algorithm based on neighbor amount and residual energy, which is called AS-NAE, is proposed to determine the sojourn site for each DCV. In addition, we build an optimization function to compute the data collection amount in an area. Then we decompose the maximization problem through penalty function and dual function mathematical method. Maximum data amount can be obtained by adjusting optimal link rates and data generation rate. Finally, we provide extensive results to evaluate the effectiveness of proposed algorithms and optimization function.

The rest of the paper is organized as follows. Section 2 studies the related work about wireless energy transfer and mobile data collection. System architecture and algorithms of network partition, starting point and anchor selection are introduced in Section 3. In Section 4, we design an optimization function to achieve maximum data amount by adjusting data rate and link rate of each node. Besides, optimal energy threshold for each node is also included. Section 5 provides extensive results for network performance. Future works and conclusion are derived in Section 6.

## 2. Related Work 

### 2.1. Energy Replenishment for WRSN

WET technology provides a new way to supply stable, high-density, sustainable energy for nodes rather than collecting energy from the environment. Powercast [17] has developed products that transmit energy to nodes at a relatively short distance. Peng et al. [18] used Powercast’s chargers to extend the network life by constructing a charging queue based on the greedy algorithm. In [19], the authors first calculated the best sampling rate according to the average energy supplement rate. Then, they designed an algorithm of calculating each node sampling rate. The main indicators of algorithm were influenced by the battery status in real-time. In [20], the battery status of each node could get through energy aggregation protocols accurately and authors designed the vehicles allocation algorithm for scheduling multiple vehicles charging nodes in a network. In [21], the author only used a vehicle to charge all nodes in the network based on the near-optimal method. However, since nodes close to static anchors may transmit and receive a large number of data packets, these nodes consume more energy than others, and the energy efficiency is relatively low. Thus, the use of a vehicle for both wireless charging and data collection could significantly improve the energy efficiency. It could maximize network practical to charge nodes with less energy in the network [15]. If the network range is larger relatively, using one vehicle is apparently not enough to support the entire network running continuously in a long time. An allocation algorithm was used to allocate each charging node to multiple vehicles in the network to minimize the number of vehicles and operate network continuously [22,23]. In addition, the energy density gathering from surrounding environment is insufficient and uneven. The cluster head nodes need to consume large amount of energy for data collection and transmission, cluster heads will quickly run out of energy. Based on this, Wang et al. [24] proposed a new scheme that combined solar energy and wireless charging. It could perfectly solve different energy demand of the cluster head nodes and other nodes through experimental verification.

Although the node around the anchor will have higher average energy consumption, energy consumption and energy threshold of each node are assumed to be consistent or little different in the above works. Thus, we will take the different energy consumption rate and energy threshold of all nodes into consideration to extend the sensor nodes lifetime.

### 2.2. Mobile Data Collection

Thus far, there have been many methods used for data collection from the initial static sink node, dynamic sink node to mobile collector [25,26]. Most of these works are divided into three steps. First, they choose proper nodes as anchor points. The second step is to design the efficient transmission link within the cover range of cluster. It is used to make the cost and time from nodes to anchors less and shorter. Third, they project the shortest movement of all anchors to reduce the collector’s energy consumption. To achieve high link utilization and high throughput, Xiong et al. [27] developed an efficient flow control scheme to receive data at different rate. In [15], the authors used a vehicle to collect data and recharge node. Although using the vehicle relieved the death node amounts in the network, data needed to wait for a long-time to be collected, increasing the data collection delay due to the charging time cannot be neglected. To solve this problem, Wang et al. [16] used a data collector gathering data from anchor point and a charging vehicle charging nodes in the network. It could greatly reduce the waiting time for data to be delivered. The problem of reducing the data collection latency has been studied for long time. Liu et al. [28] proposed a semi random circle routing for mobile sink to ensure the data delay acceptably. In [29,30], the problem was considered as Traveling Salesman the Problems (TSP) with Neighborhoods. Besides, they designed a heuristic algorithm reducing the tour path of collector and data collection delay. Yang et al. [31] used heuristic algorithm to design the collection path of the collector to balance the traffic load in the multi-hop transport. In [32], the authors proposed using the mobile base stations to collect data which were similar to data collectors and designed the optimal mobile path for mobile base stations. Zhao et al. [33] joint considered the collector’s movement control and space division multiple access (SDMA) technology to balance the movement of the collector consumption and data collection amount with SDMA techniques. Zhao [34] leveraged optimized distributed algorithms to maximize network data collection. Reducing data collector tour length is very efficient by reducing the number of the anchor point, namely the number of sites in the process of collecting data. Chen et al. [35] put forward an efficient algorithm of energy-efficient data collection. Although it largely prolonged the network life span, the tour length of data collection period is extended. In [36], a collaborative virtual environment was carried out to integrate information from different vehicles. Besides, in [37], Controlled Data and Interest Evaluation in Vehicular Named Data Networks Interest was proposed to control data flooding or broadcast storm. It could reduce data packet copies and minimize the overall interest satisfaction delay. Similarly, Ahmed et al. [38] used an Autonomous Underwater Vehicle (AUV) to maximize collection data packets. They took time-sensitive and information importance into consideration to reduce overlapping information. Chen et al. [39] put forward an efficient algorithm of energy-efficient data collection. Although it largely prolonged the network life span, the tour length of data collection period is extended.

In the above work, although most of the works take into account energy consumption of the data collector in data gathering process, they do not think about the choice of sojourn site to reduce energy consumption. Therefore, in this article, we consider the mobile site selection and TSP path for all sites. At the same time, the node energy and the node numbers in a cluster are taken into account to minimize data transmission delay.

## 3. Network Model and Data Collection 

The network model and some data collection scheme are presented in this section. In this section, we do data collection in three steps. The first step is to divide the network into several parts. Using multiple WCVs and DCVs is essential when the network area is large. The second step is to select the appropriate starting point of the vehicles. Assuming the two types of vehicles start from the same location in the smaller area after segmenting the network. It is effective to reduce the movement energy consumption of the two types of the vehicles by finding suitable starting point in each region. Finally, we should determine anchor points and the shortest moving distance of DCV in each small area, namely the stop sites and tour length for DCV. Here, the anchor point is the sensor node that collects packets from other nodes. DCV stays at the anchor point and collects packets that are passed to the anchor point. In addition, the selection of anchor points is dynamic. Each region will reselect the anchor point to avoid the hotspot problem every fixed cycle. Then, the TSP path of all anchors is constructed to reduce the movement energy consumption and data collection delay.

### 3.1. Network Model

The whole network is composed of sensor nodes uniformly distributed which can be modeled as an undirected graph G=(V,E), where *V* is the number of rechargeable nodes in the whole network. *E* is the set of undirected edges, i.e., E={E|(i,j)∈E,i,j∈V}. Both *V* and *E* in *G* are finite nonempty sets. Each link of *E* is an ordered pair of two different vertices in *V*. In [40], if two nodes *i* and *j* could transmit data to each other with the power lower than the maximum transmission power, these two nodes were connected to each other. The maximum transmission range of each node depended on its maximum transmission power. Nodes could receive data from others only in the transmission range of neighbors. We assume that all links were bi-directional. The network has no loops and no two nodes of its links join the same pair of vertices. In addition, the whole network is a connected graph, that is, any two nodes are connected at least one path. There are no independent nodes in the entire network. For independent nodes existing, we refer to the problem as “local maximum” [40,41].

Figure 1 gives a vivid description of the whole network. The rechargeable sensor nodes can be divided into two classes: normal nodes and anchor points. Anchor points are the nodes collecting data from others. The normal nodes can transmit their own data to anchor points with multiple techniques. The base station is located at the central position of the whole network, which is used to process all data packets and charge the vehicles to maintain the continuous operation of the whole network. WCV and DCV are respectively the energy transmitter and data collector in the network. DCV collects data from each anchor point during the periodic tour of the entire network. Subsequently, DCV uploads the collected data to the base station. The charging request nodes are sorted according to the charging rules. Hence, the charging queue is constructed for WCV. Then the WCV tours the whole area to charge node one by one based on charging queue. Once WCV and DCV have lower energy, they return to the base station to replenish their energy.

### 3.2. Adaptive Network Partition

Because using one WCV and DCV cannot cover effectively the entire wide range network, multiple vehicles are employed to charge nodes and collection data. To handle the complex scheduling problem of multiple vehicles in large-scale networks, we propose to divide the network into several parts and distribute a WCV and a DCV in each small region. The method of zoning regulation can relieve the vehicles schedule problem. In addition, the problem of vehicles schedule is transmitted to network partition and the path planning of single type vehicle in a sub-region. We assume that the number of WCVs and DCVs is the same in the large-scale network. We can use Equation (13) in [16] to calculate the number of vehicles needed. Through the data generation rate is dynamic, we can model the data generation process as Poisson process to estimate the vehicles number. Since the vehicles number *m* in the network is known, the number of network partitions *c* can get through *c* = *m*. Hence, there is a WCV and a DCV in each sub-region to ensure energy transmission and data collection. In addition, we can get the cartesian coordinates of nodes based on [15,42]. The process of dividing network is as follows.

- Carry out the first partition. This is similar to the method in [16] to divide the network. However, we divide the square network into *c* parts uniformly not using K-means method [16]. The other difference is the selection of the initial center. After the region is divided into *c* parts, we select a node in each part as the initial center point. The initial center points firstly are the node closest to the center location of each region, instead of the node with minimal energy. In addition, we calculate the distance *d’* and the shortest routing hop *h’* from each node to all central points. ${d^{\prime }}_{i}^{j}$ means the distance between node *i* and center point *j*, where i∈N,j∈c. Then we sort the distance values ${d^{\prime }}_{i}^{j}$ of node *i* in an increasing order. $D_{i}^{j}$ means the serial number of the distance ${d^{\prime }}_{i}^{j}$ from node *i* to the center point *j*. When $D_{i}^{j}$ = 1, it means ${d^{\prime }}_{i}^{j}$ is the minimum distance. When $D_{i}^{j}$ = *c*, it means ${d^{\prime }}_{i}^{j}$ is the maximum distance. In a similar way, ${h^{\prime }}_{i}^{j}$ is denoted as the routing hop between node *i* to center point *j*, where i∈N,j∈c. We sort the *c* numbers in an increasing order. $H_{i}^{j}$ means routing hop serial number from nodes *i* to center point *j*.
- Carry on the second partition. We compute the weight $W_{i}^{j}$ of each node *i* to *j* firstly. Here, $W_{i}^{j}=\alpha D_{i}^{j}+\beta H_{i}^{j},\alpha +\beta =1,0\leq \alpha ,\beta \leq 1,\forall i\in N,\forall j\in [1,c]$. *α* and *β* are defined as the proportions of distance priority and routing hop priority. Define $\vartheta =\frac{\alpha }{\beta }$ as the ratio of *α* and *β*. If ϑ→∞, our algorithm can be regarded as considering routing hop only. On the contrary, our algorithm can be transformed as taking distance into account merely when ϑ→0. In this scenario, we joint consider distance and routing hop equally to make ϑ=1. Subsequently, we select the smallest $W_{i}^{j}$ as a result of partitioning and assign the node *i* to the *j*th region. Loop this process until all nodes in the network are partitioned. The adaptive network partition algorithm with twice-partition is shown in Algorithm 1.
**Algorithm 1** A Twice-Partition Algorithm Based on Center PointsInput: sensor nodes *N*, center points numbers *c*.Output: min {$W_{i}^{1},\ldots ,W_{i}^{c}$}.Compute distance ${d^{\prime }}_{i}^{j}$ and shortest routing hop ${h^{\prime }}_{i}^{j}$ between node *i* to center point *j*, where i∈N,j∈c.Sort $\left\{{d^{\prime }}_{i}^{1},\ldots ,{d^{\prime }}_{i}^{c}\right\}$ and $\left\{{h^{\prime }}_{i}^{1},\ldots ,{h^{\prime }}_{i}^{c}\right\}$ in an increasing order.$D_{i}^{j}$ means distance serial number and $H_{i}^{j}$ means routing hop serial number of node *i* to center point *j*.While N≠∅  Compute $W_{i}^{j}=\alpha D_{i}^{j}+\beta H_{i}^{j},\alpha +\beta =1,0\leq \alpha ,\beta \leq 1,\forall i\in N,\forall j\in [1,c]$.  $W_{i}^{j}=$ min {$W_{i}^{1},\ldots ,W_{i}^{c}$}, assign the node *i* to the *jth* region.  *N* = *N* − *i*.End

Besides, acquiring the distance and routing hop for every node needs at least |Nc|. Sorting time complexity for every node is O(|c|⌈log|c|⌉). In addition, there are *N* sensor nodes, which means we should compute |Nc|⌈log|c|⌉. Finally, to compute *W_i_* and find the minimum value in $W_{i}^{1},\ldots ,W_{i}^{c}$, the time complexity is |c| and O(|c|⌈log|c|⌉) respectively. Thus, the overall complexity of this algorithm is O(|Nc|⌈log|c|⌉).

**Figure 2:**
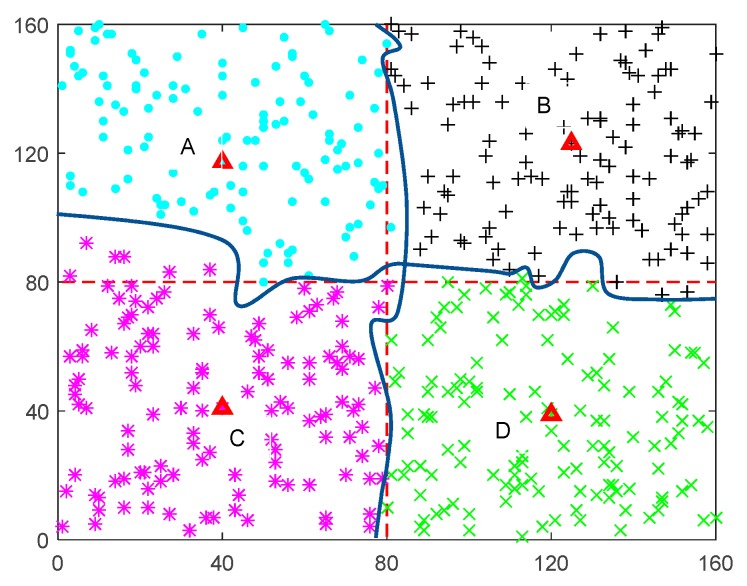
Network partition based on center points.

Figure 2 shows the results of network partition. The red dotted lines equally divide the network into four parts: A, B, C, D. That means the first partition. In addition, the red triangle points indicate the initial point of each region. Then we run the second partition based on these center points. The same shape and color symbols represent the node belong to a partition after operating partition algorithm. The blue lines represent the results of final partition. Obviously, some nodes belonging to area A before are assigned to the B and C regions.

### 3.3. Vehicles Starting Point

Once the network partition is determined, we need to ensure the starting point position of two types of vehicles within each region. It is important to get the position with the minimum sum of distance and routing hop to all nodes in a part, which can reduce the energy consumption of movement and data transmission. The vehicle starting from the position will reduce the movement consumption while touring the whole network. In addition, if the node to others has minimum routing hops sum, the node is likely chosen as an anchor or it is close to an anchor. Once we choose the node position as staring point, it can reduce energy consumption on transmitting and receiving data packets to a large extent. Besides, it can decrease the energy consumption for moving the position to collect data.

In the experimental scenario, the two types of vehicles share one starting point in a sensing region after partition. In the initial state, vehicles will be deployed in the starting position. In addition, vehicles have an energy threshold to make sure that they can move back to base station to replenish energy. When a data collection cycle or charging cycle is finished, vehicles will move back to starting position waiting for the next round. Thus, the vehicles always starting from the starting point to gather data or charge nodes. The starting point of WCV was expounded in [16]. After calculating the sum of the distances between each node to other nodes in the cell, the node with minimal distance was selected as the starting point of the WCV. In [43], the authors used the dynamic sink nodes and the mobile base station to collect data. The position of sink nodes changed with position of the base station. Although it did not use a mobile data collector to collect data, this method was similar to select the starting point of vehicles. We take an area after partitioning as an example in the statement. Now we briefly explain the process of choosing starting point.

Initially, we compute $S_{k}=\sum_{i=1}^{n_{r}}\|n_{i}-\mu_{k}{\|}^{2}$, where $\|n_{i}-\mu_{k}{\|}^{2}$ is squared distance between nodes *i* and *k*. $n_{r}$ is the number of nodes in an area, $\mu_{k}$ represents the *k*th node in this region and $k\in [1,n_{r}]$. Each node has a sum value of squared distance, *S_k_*, and we sort these values in an increasing order for all nodes in a cell. $S_{k}^{\prime }$ is the ordinal number of the node *k*. Then, we calculate $H_{k}=\sum_{i=1}^{n_{r}}\left\|h_{i}^{k}\right\|$, where $h_{i}^{k}$ is the routing hop from node *i* to *k*. Similarly, the routing hop of all nodes is sorted in an increasing order. $H_{k}^{\prime }$ is the ordinal number of the node *k*. Finally, the weight of node *k* is computed. Here,$W_{k}=\alpha S_{k}^{\prime }+\beta H_{k}^{\prime }$, where $\alpha +\beta =1,0\leq \alpha ,\beta \leq 1,\forall k\in [1,n_{r}]$. Similar to the setting in the network partition, we can arbitrarily change the percentage of distance and routing in the selection of the start point. The *W_k_* of all nodes in the region is sorted. The location of the nodes with the minimum *W* value is selected as the starting point of the two types of vehicles. Now, the point has the minimum distance and routing hops to all nodes. It can reduce energy consumption on transmitting and receiving data packets because the position is likely near to anchor. Specific algorithms are shown in Algorithm 2.
**Algorithm**
**2** Starting Point of Vehicles Selection AlgorithmInput: sensor nodes *n_r_* in a cell.Output: min {$W_{k}$}, $\forall k\in n_{r}$.Compute distance $S_{k}=\sum_{i=1}^{n_{r}}\|n_{i}-\mu_{k}{\|}^{2}$ for node *k*, where $\forall k\in n_{r}$.Compute routing hop $H_{k}=\sum_{i=1}^{n_{r}}\left\|h_{i}^{k}\right\|$ for nodes *k*, where $\forall k\in n_{r}$.Sort $\left\{S_{1},\ldots ,S_{n_{r}}\right\}$ and $\left\{H_{1},\ldots ,H_{n_{r}}\right\}$ in an increasing order.$S_{k}^{\prime }$ means distance serial number and $H_{k}^{\prime }$ means routing hop serial number of node *k*.Compute $W_{k}=\alpha S_{k}^{\prime }+\beta H_{k}^{\prime }$, where $\alpha +\beta =1,0\leq \alpha ,\beta \leq 1,\forall k\in [1,n_{r}]$.$W=\min\{W_{1},\ldots ,W_{n_{r}}\}$, assign starting point to the position of node with *W*.

The time complexity in computing distance and routing hop for every two nodes is both $O(|n_{r}{|}^{2})$. Now, every node has a distance value and routing hop number to others. Sorting these values for all nodes is $O(|n_{r}|\lceil \log|n_{r}|\rceil )$. Finally, the time complexity is $O(|n_{r}|\lceil \log|n_{r}|\rceil )$ to choose the minimum value in $\left\{W_{1},\ldots ,W_{n_{r}}\right\}$. Thus, the time complexity for the algorithm is $O(|n_{r}{|}^{2})$.

### 3.4. Adaptive Anchor Point Selection

Next, we should determine the DCV’s sites during the data collection process. To design an efficient data collection scheme, the anchor selection algorithm should satisfy two requirements. First, the energy of anchor node should be as much as possible. Because the anchor and its nearby nodes need frequent transmit plenty of data packets consuming more energy compared to the other nodes. Second, anchor selection algorithm should select a subset of nodes while maximizing the amount of data collected and minimizing the anchor amounts to meet the delay requirement of nodes.

There are some methods to determine anchor points. In [16], by arranging a series of circles to cover the whole network, the center of the circle was chosen as the data collection anchor point. However, there are no constraints on sensor energy and amounts in a cluster. These nodes near to anchors expend more energy than others due to data transceiver. In [26], the authors computed weighted average of each node through the nodes amounts within the *k* hops in the network and selected the node with maximum weighted average value as the anchor. Although the scheme considers both energy and sensor amounts in a cluster, there is a drawback. In a cluster, when the energy of some nodes is quite high and some nodes are very low, the energy factor makes little sense for anchor selection. In [15], the method was relatively simple, mainly considering the energy limit at the anchor point. Its first step was constructing the energy queue by the least energy of each sensor’s neighbors within *k* hops. Then the method selected the node with max value in energy queue as the anchor. The node amounts within *k* hops are ignored, which can cause hotspot problem due to transmit and receive extensive data packets. In [44], they just divided the network into grid. Anchor points could be uniformly distributed on grid intersections. Another selection method was to directly select some nodes covering the entire network as anchors to collect data [45]. These two methods do not consider energy or nodes amount, which increases energy consumption and data latency.

Our goal is to select a set of nodes in the network covering the whole network and its tour length is within a bounded threshold *L_b_*. Thus, we propose an anchor selection algorithm called AS-NAE. Anchor selection algorithm is as shown in Algorithm 3.
**Algorithm**
**3** Anchor Point Selection Algorithm.Input: sensor nodes *n_r_* in a cell, connected matrix *X*.Output: Anchor Point list A.Compute node amount within *k* hops for nodes *i*
$N_{i}=\sum_{j=1}^{n_{r}}X_{ij}$, Compute the least energy of nodes *i* within *k* hops $BATT_{i}=\{\min\{C_{b_{j}}\}|dist(i,j)\leq k,\forall X_{ij}=1\}$.Sort $\left\{N_{1},\ldots ,N_{n_{r}}\right\}$ and $\left\{BATT_{1},\ldots ,BATT_{n_{r}}\right\}$ in a decreasing order.$NUM_{i}^{\prime }$ means nodes amount serial number and $BATT_{i}^{\prime }$ means energy serial number of node *i*.Compute $W_{i}=NUM_{i}^{\prime }+BATT_{i}^{\prime }$ and Sort $\left\{W_{1},\ldots ,W_{n_{r}}\right\}$ in an increasing order, denote as *N_s_*.Initialize node index *i* = 1, *k* = 1, *j* = | *N_s_* |.While $n_{r}\neq \emptyset$  Obtain *i*th element in *N_s_* and insert to *A*;  For *k* from 1 to *j*  If *X_ik_* > 0, $N_{s}\leftarrow N_{s}-k$ and set *k* as descendant of *i*; End if  End for  $N_{s}\leftarrow N_{s}-i$;End whileObtain anchor point list *A* from above.While *true*Compute the shortest migration tour *L_tsp_* through sensors in *A*.If $L_{tsp}\leq L_{b}$, break;Else Remove anchors with the largest *W_i_* from *A*; End ifEnd while

Initially, we need to construct a connected matrix *X* to show whether the nodes in the region are connected with other nodes within *k* hops, as shown in Equation (1). The cluster size of *k* has an important effect on energy consumption and data collection latency. Choosing a larger *k* can reduce the number of stay site of DCV in touring network and decrease mobile energy consumption. Accordingly, an anchor will cover more nodes for data collection and energy consumption will increase. When the sojourn time of each site is constant, data collection delay will be increase too. On the other hand, a small *k* may produce more anchors and the tour length of DCV may be increasing. When the data collection period of *T* is unchanged, collection delay will higher. Therefore, choosing the right *k* has a crucial impact on the entire network. For simplicity, we set *k* equal to 3, which is just equal to $\frac{k_{\max}}{3}$. *k_max_* is the max routing hop for two nodes in a cell.

(1) $$X_{ij}=\{\begin{array}{ll} 1 & \mathrm{if}\;i\;\mathrm{and}\;j\;\mathrm{is}\;\mathrm{connected}\;\mathrm{within}\;k\;\mathrm{hops}\\ 0 & \mathrm{if}\;i\;\mathrm{and}\;j\;\mathrm{is}\;\mathrm{not}\;\mathrm{connected}\;\mathrm{within}\;k\;\mathrm{hops}\\ 0 & \mathrm{if}\;i=j \end{array}$$

The anchor selection is directly related to the energy and quantity of neighbors in the *k* hops. For a set of nodes *n_r_*, $N_{i}$ is expressed the number of nodes within *k* hops, where $N_{i}=\sum_{j=1}^{n_{r}}X_{ij}$ $\forall i\in [1,n_{r}]$. Then, we construct a queue *NUM* in a decreasing order through *N_i_*. Now, $NUM_{i}^{\prime }$ is the ordinal value of node *i* in queue *NUM*. *BATT* is the set of the least energy of each sensor’s neighbors within *k* hops, that is $BATT_{i}=\{\min\{C_{b_{j}}\}|dist(i,j)\leq k,\forall X_{ij}=1\}$, where *C_b_* is residual energy of node. Similarly, $BAT{T_{i}}^{\prime }$ is the ordinal value after ranking. Thus, set $W_{i}=NUM_{i}^{\prime }+BATT_{i}^{\prime }$ and rearrange *W_i_* in a decreasing order for all nodes. The queue is denoted as *Q*. Then we select the first value as the anchor point removing the nodes within *k* jump connected in Q, namely remove the node where the value of the row corresponds to 1 in connectivity matrix *X*. This process is repeated until the queue *Q* is empty. At this point, the anchor queue *A* is the anchor point selected in the network. The TSP path of all anchor points is then computed. If $L_{tsp}\leq L_{b}$, the anchor queue is the calculated just queue *A*. Otherwise, the node with the largest *W_i_* is removed until $L_{tsp}$ less than *L_b_*.

Obviously, obtaining the connectivity matrix of the network requires at least $k|n_{r}{|}^{2}$. Computing amounts of neighbors of each node within *k* hops needs $\left|n_{r}\right|$. Then, the sorting time complexity is $O(\left|n_{r}\right|\lceil \log\left|n_{r}\right|\rceil )$. Similarly, computing the minimum battery energy for each node’s *k* hops is $|n_{r}{|}^{2}$, and the time complexity of sort the queue is $O(\left|n_{r}\right|\lceil \log\left|n_{r}\right|\rceil )$. Finally, the queue *Q* queue is sorted with a time complexity of $O(\left|n_{r}\right|\lceil \log\left|n_{r}\right|\rceil )$. Suppose that the number of anchor points is |S|. Each time we need to compute the shortest path of anchor point with the time complexity is $O(|S{|}^{2})$. Thus, the overall complexity of the anchor selection algorithm is $O({\left|n_{r}\right|}^{2})$.

**Figure 3:**
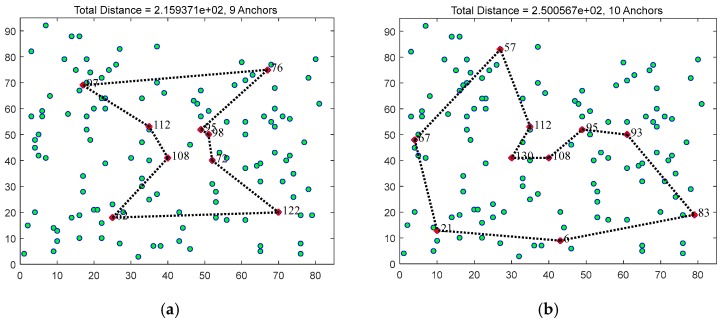
An example of anchor selections with 130 nodes under different touring bound *L_b_*: (**a**) the total distance is 251 m and 9 nodes are chosen to be anchors with threshold *L_b_* = 250 m; and (**b**) there are 10 anchors and the shortest tour length is 250 m with threshold *L_b_* = 300 m.

Next, we give an example of anchor selection algorithm. The 130 nodes are uniformly distributed in the square region of the (85,95) and the energy of the same nodes in the two figures remains the same at the initial stage. The number of data collection hop count *k* is 3. Then, the shortest travel path of DCV is obtained by using the TSP algorithm. Figure 3 shows the anchor point selection at different travel length thresholds *L_b_*. Figure 3a shows that the algorithm chooses 9 anchors when $L_{b}=250\;m$ and the shortest tour length is 215 m. Figure 3b indicates there are 10 anchors and the tour length is 250 m while $L_{b}=300\;m$. Node with a red tag indicates the selection point anchor and black dotted line represents the moving path of DCV. In addition, the coordinate of center points position in area C is approximately (40,40) from Figure 2. Figure 3 shows the anchors in area C. It is obvious that the node with serial number 108 is both chosen as anchors within different tour length threshold. The coordinate of node 108 is close to (40,40). Thus, we can say that center point is likely to be anchor or nearby node of anchors. Through experimental verification in Section 5.1, the starting position of vehicles is close to center position in a region. Thus, the starting position is very close to anchors or nearby nodes of anchors. This can reduce energy consumption on vehicle moving and data forwarding.

Although the energy of anchors and its nearby nodes are higher compared to other nodes, they will consume more energy for data forwarding and generating. In [46], authors proposed a new deployment strategy with different node density to avoid energy holes. In this paper, we rerun the anchor selection algorithm after fixed data collection cycle. The nodes with higher energy are likely to be chosen as anchors, we can relief hotspot problem through this method.

## 4. Performance Optimization

After the anchor points in the network are determined and the tour through all anchors has been constructed, the remaining question is how to collect more data from the nodes. We can translate the problem into a data collection maximization problem based on flow-level network model. Considering a network with *n_r_* nodes and *A* data collection anchor points, DCV periodically visits the entire sensing area from the starting point position to the original point finally. Once the residual energy of vehicle is lower than its threshold, DCV immediately switches back to the base station to replenish the energy. DCV collects data at each anchor sojourning a fixed time, i.e.,$\tau_{a}$. In this process, other nodes forward data to the anchor point in a multihop manner and the anchor transmits data to the DCV in a single hop mode. After that, DCV moves to the next anchor for data collection.

**Figure 4:**
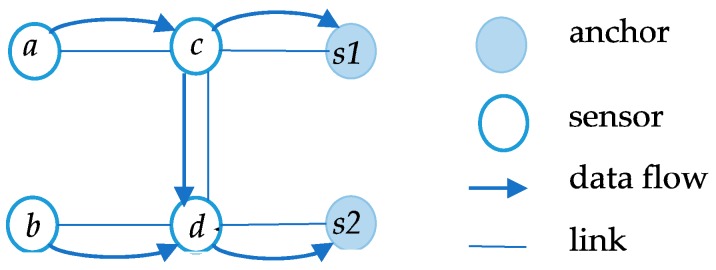
Data transmission model.

As shown in Figure 4, when the DCV stays in the anchor at *s1*, node *a* can forward packets along the link path {a,c,s1}. However, when the DCV stays in the *s2*, forwarding path from *a* is {a,c,d,s2}. When DCV is at different anchor, the packets transmission path of same node will be not the same. In addition, some nodes in the network can receive and transmit data packets at the same time in a real scenario. Thus, how to distinguish the functions of these nodes becomes the most important issue. In other words, we should recognize the direction of each link and determine the role of every node which represents sender or receiver to the other nodes. Thus, we use parents set $P_{i,a}$ and children set $C_{i,a}$ to distinguish different functions of each node, where $P_{i,a}$ represents presents set of node *i* while DCV is sitting at anchor *a*. Accordingly, $C_{i,a}$ represents the children set of node *i* when the data collection vehicle stops at anchor *a*. Importantly, the parents nodes and children nodes can communicate directly with the node *i* without forwarding other sensor nodes. In Figure 4, parent set $P_{c,s1}$ of node *c* is {s1} and child set $C_{c,s1}$ is {a} when DCV is located at *s1*. However, when DCV sojourns at *s2*, $P_{c,s2}=\{d\}$ and $C_{c,s2}=\{a\}$. Thus, the parent set and child set of the same node may be different if DCV is located at different anchor points. The entire network can be seen as a directed acyclic graph (DAG) through seeing each anchor as an end point. Modeling structured sensors as a network to study and is always using DAGs [19]. In [40], the parent node set of *i* were the neighbors that are close to the sink nodes. In the sensing area of the node, it mainly compared the distance between the node and anchor point and the neighbor node to the anchor point to get the direction of the link. Authors modeled a DAG by constructing the weight of outgoing edges of a node to the reciprocal of its residual energy and then using Dijkstra’s algorithm to produce a directed tree toward anchors [15]. In this paper, we make the nodes directly connected with the anchor as the anchors’ children set nodes. Then we get children set of others through its parents set. Repeat this process until the parents and children set are found. Based on this, we consider the network as node-exclusive interference model. A node cannot communicate two or more nodes at the same time. For each node in the area, there are three cases for energy consumption, i.e., $e_{r},e_{t},e_{s}$. They represent average energy consumption on receiving, transmitting and sensing a data packet. The related parameters are shown in Table 1.

### 4.1. Formulate Problem for Mobile Data Gathering

Our goal is to maximize the amount of data collected by DCV. The problem is closely related to the data rate of each node, which is subject to the link rate, the energy balance and link capacity. Therefore, we introduce the utility function *U_i_*(*·*) to show the influence of the data rate of the node *i* on the performance of the whole network data collection. Obviously, the utility function is an increasing function due to the vehicle can collect more data with data generation rate increasing. Besides, each node has a different data rate in each anchor, the superposition process makes the value of utility function higher. Accordingly, we have reason to show that the data rate of the utility function with respect to node *i* is an increase, strictly concave and twice-differentiable. Now, the optimization problem is as follows.

(2) $$P1:\;\max_{r,f}\sum_{i}U_{i}(\sum_{a}r_{i}^{a})$$

subjected to

(3) $$\sum_{c\in C_{i,a}}f_{ci}^{a}+r_{i}^{a}=\sum_{j\in P_{i,a}}f_{ij}^{a}$$

(4) $$\tau_{a}\Phi (f,r)\leq C_{b}$$

(5) $$0\leq \sum_{a\in A}f_{ij}^{a}\leq \pi_{ij}$$

where

(6) $$\forall i\in n_{r},\forall a\in A,\forall c\in C_{i,a},\forall j\in P_{i,a}$$

(7) $$\Phi (f,r)=\sum_{j\in P_{i,a}}f_{ij}^{a}e_{t}+\sum_{m\in C_{i,a}}f_{mi}^{a}e_{r}+r_{i}^{a}e_{s}$$

(8) $$\tau_{a}=\frac{T-L_{tsp}/v}{\left|A\right|}$$

These constraints can be explained as follows. Each node’s data packets have two sources that receive packets from other nodes and its own generated packets. The first set of Constraint (3) indicates the outgoing traffic flow is equal to the amount of data received by other nodes and generated. Constraint (3) ensures that the output and input stream are balanced at each node corresponding to the parents and children set of each node. Equation (4) ensures that the energy consumed by nodes is not greater than the residual energy of nodes in one cycle. The energy consumed includes the generation, reception and transmission of packets, which guarantees node survival to the maximum extent. Since each link is affected by bandwidth and channel capacity, in this paper, we set the maximum link rate for each link. The third constraint in Equation (5) indicates that the capacity in a link should be below the maximum capacity.

Here, we use the similar optimization function in [15,26]. Although the objective function is same, we add data sensing energy consumption in the constrain condition compared to [15]. The energy consumption on sensing data has a great influence on node lifetime. There is no doubt that it will affect the setting on node energy threshold. There are several differences in the constrain condition compared to [26]. First, we do not gather energy from the surrounding environment, so the node battery constraints subtract the part of gathering solar energy. Besides, the sojourn time in each anchor are set fixed in data collection cycle, which simplify the calculation process on data rate and link rate.

As mentioned earlier, the utility function is a strict concave function for the data generation rate at all anchor points of node *i*. Our aim is to obtain the data rate $r_{i}^{a}$ and link rate $f_{ij}^{a}$ at each anchor point. Since the utility function $U_{i}(\sum_{a}r_{i}^{a})$ is independent of the link rate. Therefore, we only consider the relation between data rate and utility function. To obtain the link rate at a certain anchor point, it is not sufficient by only using $\sum_{a}r_{i}^{a}$. Thus, we need to obtain the relation between $r_{i}^{a}$ and the utility function. However, problem P1 is not strictly concave for $r_{i}^{a}$ because of the linearity of $\sum_{a}r_{i}^{a}$. To solve this problem, we use the penalty function method adding a quadratic term $-\sum_{i\in n_{r}}\sum_{a\in A}\frac{1}{2c_{i}}{\left(r_{i}^{a}-y_{i}^{a}\right)}^{2}$ to the original objective function so that the augmented objective function is strictly concave for $r_{i}^{a}$. Now, $c_{i}$ is the positive number chosen for each node and $y_{i}^{a}$ is the additional variable for $r_{i}^{a}$. The procedure for calculating the objective function is as follows. First, we fix $y_{i}^{a}$ according to its initial value. Then, calculate the data rate and link rate for each node. We assume that ${r_{i}^{a}}^{*}$ is the optimal data generating rate of the node *i* at anchor *a*. In addition, set $r_{i}^{a}={r_{i}^{a}}^{*},y_{i}^{a}={r_{i}^{a}}^{*}$ and repeat the above process until $y_{i}^{a}$ convergence. We can get the optimal data rate and link rate finally.

### 4.2. Lagrange Dual and Sub-Problem for Data Rate and Link Rate

Lagrange multiplier method is a common and convenient method for solving optimization problems. We introduce the Lagrange multiplier ς,λ for Constraints (3) and (4). Then, we can obtain the Lagrange of the augmented objective function as follows.

(9) $$\begin{array}{l} L(r,f,\varsigma ,\lambda )=U_{i}(\sum_{a\in A}r_{i}^{a})-\sum_{a\in A}\frac{1}{2c_{i}}{\left(r_{i}^{a}-y_{i}^{a}\right)}^{2}\\ -\sum_{a\in A}\varsigma_{i}^{a}(\sum_{c\in C_{i,a}}f_{ci}^{a}+r_{i}^{a}-\sum_{j\in P_{i,a}}f_{ij}^{a})\\ -\sum_{a\in A}\lambda_{i}^{a}((\sum_{j\in P_{i,a}}f_{ij}^{a}e_{t}+\sum_{m\in C_{i,a}}f_{m,i}^{a}e_{r}+r_{i}^{a}e_{s})-\frac{C_{b}}{\tau_{a}})\\ =U_{i}(\sum_{a\in A}r_{i}^{a})-\sum_{a\in A}\frac{1}{2c_{i}}(r_{i}^{a}-y_{i}^{a}{)}^{2}-\sum_{a\in A}\left(\varsigma_{i}^{a}+e_{s}\lambda_{i}^{a}\right)r_{i}^{a}\\ +\sum_{a\in A}(\sum_{j\in P_{i,a}}(\varsigma_{i}^{a}-\lambda_{i}^{a}e_{t})f_{ij}^{a}-\sum_{m\in C_{i,a}}(\lambda_{i}^{a}e_{r}+\varsigma_{i}^{a})f_{mi}^{a}) \end{array}$$

The dual function of the objective function is as follows.

(10) $$\underset{\begin{array}{l} \varsigma \geq 0\\ \lambda \geq 0 \end{array}}{\min}D(\varsigma ,\lambda )=\min_{\begin{array}{l} \varsigma \\ \lambda \end{array}}\max_{\begin{array}{l} r\\ f \end{array}}L$$

It is obvious that the dual function can be decomposed into two subproblems, namely the node rate control subproblem and joint scheduling and routing subproblem. Formula (9) divides the target problem into two parts, namely the node rate control problem (Formula (11)) and the routing problem (Formula (12)).

(11) $$L_{1}(r,\varsigma ,\lambda )=U_{i}(\sum_{a\in A}r_{i}^{a})-\sum_{a\in A}\frac{1}{2c_{i}}(r_{i}^{a}-y_{i}^{a}{)}^{2}-\sum_{a\in A}\left(\varsigma_{i}^{a}+e_{s}\lambda_{i}^{a}\right)r_{i}^{a}$$

(12) $$L_{2}(f,\varsigma ,\lambda )=\sum_{a\in A}(\sum_{j\in P_{i,a}}(\varsigma_{i}^{a}-\lambda_{i}^{a}e_{t})f_{ij}^{a}-\sum_{m\in C_{i,a}}(\lambda_{i}^{a}e_{r}+\varsigma_{i}^{a})f_{mi}^{a})$$

We use derivative and differential mathematical methods solving Formula (11) to maximize the function value. By using the method in [15,26], we first obtain the value of $U_{i}^{\prime }(\cdot )-\frac{1}{c_{i}}r_{i}^{a}+\frac{1}{c_{i}}y_{i}^{a}-(\varsigma_{i}^{a}+\lambda_{i}^{a}e_{s})+\sigma_{i}^{a}=0$ by using the Karush–Kuhn–Tucker (KKT) condition, where $U_{i}^{\prime }(\cdot )=U_{i}^{\prime }(\sum_{a\in A}r_{i}^{a})$ is the first derivative of $U_{i}$ and $\sigma_{i}^{a}$ is Lagrange multiplier about $r_{i}^{a}\geq 0$. According to the prerequisite $r_{i}^{a}>0$, we can infer $\sigma_{i}^{a}=0$. The data rate in each anchor is as follows.

(13) $$r_{i}^{a}=\{\begin{array}{ll} (U_{i}^{\prime }(R_{i})+m_{i}^{a})c_{i} & a\leq p\\ 0 & a>p \end{array}$$

Besides, we define that 1≤p≤|A|, $R_{i}=\sum_{a}r_{i}^{a}$ and |*A*| is anchors number in an area. We can calculate *R_i_* by using $AU_{i}^{\prime }(R_{i})-\sum_{a=1}^{A}(\frac{1}{c_{i}}r_{i}^{a}+\frac{1}{c_{i}}y_{i}^{a}-(\varsigma_{i}^{a}+\lambda_{i}^{a}e_{s})+\sigma_{i}^{a})=0$. The next step is to find the right *p*. Since *p* divides the |*A*| into two parts, where data rate is larger than 0 and equal to 0. Thus, we can use Formula (13) to find the right *p* value through dropping *p* from |*A*| to 1.

For joint scheduling and routing problem, the Formula (12) can be expressed as follows with Constraints (4) and (5).

(14) $$\begin{array}{l} L_{2}(f,\varsigma ,\lambda )=\sum_{a\in A}(\sum_{j\in P_{i,a}}(\varsigma_{i}^{a}-\lambda_{i}^{a}e_{t})f_{ij}^{a}-\sum_{m\in C_{i,a}}(\lambda_{i}^{a}e_{r}+\varsigma_{i}^{a})f_{mi}^{a})\\ =\sum_{a\in A}\sum_{j\in P_{i,a}}\left(\varsigma_{j}^{a}-\varsigma_{i}^{a}-\lambda_{j}^{a}e_{t}-\lambda_{i}^{a}e_{r}\right)f_{ij}^{a} \end{array}$$

The problem can be solved from the node with empty children set. According to the initial value of Lagrange multiplier, we can compute Lagrange multiplier and link rate with multiple iterative calculations at different anchors. It is equivalent to find the maximum *X_i_* with parent node and anchor in the collection $X_{i}=\{(j,a)|\varsigma_{j}^{a}-\varsigma_{i}^{a}-\lambda_{j}^{a}e_{t}-\lambda_{i}^{a}e_{r}>0,\forall j\in P_{ij}^{a},\forall a\in A\}$ and give the maximum link rate $f_{ij}^{a}=\frac{C_{b}}{\tau_{a}e_{t}}$. Then update the residual energy of the node and repeat the process until the collection is empty. We can use heuristic distribution algorithms in [47] to solve such problems.

When we calculate the data rate and link rate for each node, another important factor is the iteration of the Lagrange multiplier. Each node needs to update its Lagrange multipliers and send the values to the neighbor nodes directly connected to calculate the data rate and link rate of neighbor nodes. The specific iteration Formula is as follows.

For simplicity, in this paper, the initial values of the Lagrange multipliers ς,λ of all nodes are set to 1. *t* represent the number of iterations of the algorithm and ε(t)=d/(b+ct),∀d,c>0,∀b≥0 which represents the iteration step in each cycle of the multiplier, where *b*,*c*,*d* are variable parameters that adjust the iteration speed.

(15) $$\begin{array}{l} \varsigma_{i}^{a}(t+1)={\left[\varsigma_{i}^{a}(t)+\varepsilon (t)▽L(\varsigma )(t)\right]}^{+}\\ ={\left[\varsigma_{i}^{a}(t)+\varepsilon (t)(r_{i}^{a}(t)+\sum_{m\in C_{i,a}}f_{mi}^{a}(t)-\sum_{j\in P_{i,a}}f_{ij}^{a}(t))\right]}^{+}\\ \lambda_{i}^{a}(t+1)={\left[\lambda_{i}^{a}(t)+\varepsilon (t)▽L(\lambda )(t)\right]}^{+}\\ ={\left[\lambda_{i}^{a}(t)+\varepsilon (t)(\Phi (f(t),r(t))-\frac{C_{b}}{\tau_{a}})\right]}^{+} \end{array}$$

In the process of convergence, the Lagrange dual method cannot be applied directly to the original problem when $y_{i}^{a}$ is convergent. Thus, we need to recover the original value for the resulting value. The original solution is recovered by using Formula (26) in [26]. Once the original solution $\left\{{\hat{f}}_{ij}^{a}\right\}$ converges, the $f_{ij}^{a}$ is the optimal solution of the utility function.

### 4.3. Optimal Charging Threshold 

In order for the network to run continuously, we need to selectively charge each node to ensure the survival of nodes. Two problems need to be settled: (1) which nodes should be selected to be charged; and (2) how to schedule the nodes that need to be recharged. To solve the first problem, we set an energy threshold for each node. Once the residual energy of the node is below the threshold, it will send a charging request to the WCV immediately. There are many methods for charging threshold setting. In [48], they just considered the hardware energy consumption of each sensor. Thus, the charging threshold is only relevant to the maximum waiting charging time. In [16], although authors set an adaptive recharge threshold for each node, they assumed the data generation process could be modeled as a Poisson process with average rate *λ_1_*. However, because each node is inconsistent with the amount of data transceiver and amount of data rate, the energy consumption per node is a dynamic random variable that depends on the number of packets. Determining the amount of traffic per node is important for node energy consumption. Accordingly, we set a dynamically varying energy threshold to accommodate changes in network traffic. The setting of the energy threshold has a very important condition, that is, the nodes can transmit and receive data with the normal waiting for charging to ensure the continuous operation of the network. Energy consumption for node *i* is shown in Equation (16).

(16) $$P_{i}=e_{s}\sum_{a}r_{i}^{a}+(e_{r}\sum_{j\in P_{i,a}}f_{ij}^{a}+e_{t}\sum_{k\in C_{i,a}}f_{ki}^{a})+V_{d}$$

In Formula (16), the first part is sensing data consumption and the second part is energy consumption on data packet transceiver. The third part is hardware energy consumption. In the worst case, the moving time of WCV can be expressed as $t_{1}=\frac{d_{\max}}{v}$. Here, $d_{\max}$ is the distance between the two farthest nodes in the area and *v* means the speed of vehicle. After determining energy consumption of each node, the next is to compute the longest waiting time for charging request served. We use the charging request model in [48], charging request is subjected to Poisson distribution with parameter λ. The waiting time for WCV accepting the charging request is subject to exponential distribution with parameter μ. The number of requests for charging buffer is $L_{q}=\frac{\lambda^{2}}{\mu (\mu -\lambda )}$. The average waiting time for charging request is $t_{2}=\frac{\lambda }{\mu (\mu -\lambda )}$. As a result, the threshold (Formula (17)) of each node is shown.

(17) $$\begin{array}{l} \phi_{i}=P_{i}(t_{1}+t_{2})\\ =(e_{s}\sum_{a}r_{i}^{a}+e_{t}(\sum_{j\in P_{i,a}}f_{ij}^{a}+\sum_{k\in C_{i,a}}f_{ki}^{a})+V_{d})\frac{\lambda (\lambda -1)}{\mu (\mu -\lambda )} \end{array}$$

Once the node energy is below the charging threshold, the node sends energy request immediately to the WCV. After charging request is received by WCV, WCV first builds shortest tour for request nodes in charging buffer. Then, the WCV charges node in accordance with the tour path.

## 5. Performance Evaluation

In this section, we discuss the feasibility and efficiency of the mobile data collection and wireless charging scenario. Utility function is defined as $U_{i}(\cdot )=\alpha_{i}\log(\sum_{a}r_{i}^{a})$ that represents the amount of data generated by the node *i. α_i_* is the utility weight for each node *i* and higher weight has a more significant influence on the whole network. We use the network model shown in Figure 1, where *N* = 500 nodes are evenly distributed in the square area of length *L* = 160 m. All nodes transmit data at a fixed power level with the sensing range *d_r_* = 15 m. To compute the Lagrange multipliers with updating iteration step ε(t)=1/(1+100t) of each node, every node runs the same data rate and routing algorithms and communicate neighbor node to exchange its multipliers. Because each region of network can be adjusted via the network partition. Therefore, our scheme is also applicable to large-scale network. Other parameter settings are shown in Table 2.

### 5.1. Performance Analysis for Starting Point and Data Rate Algorithm 

**Figure 5:**
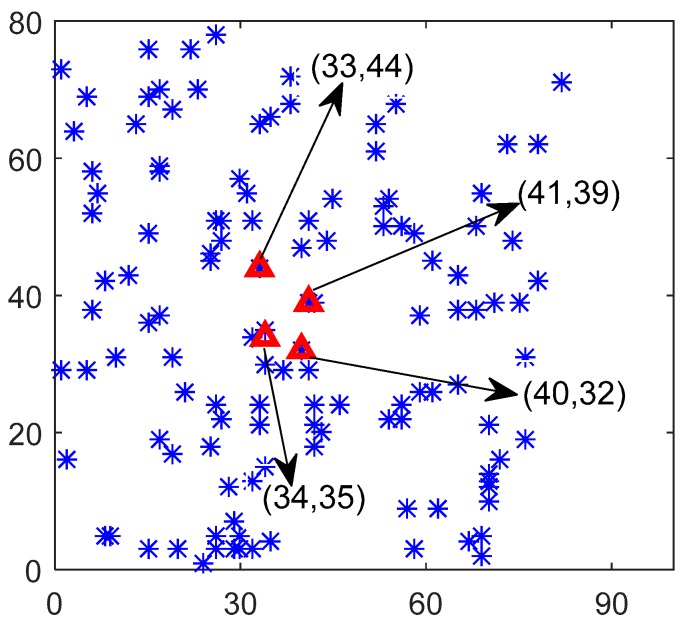
Selection of Starting Point for Vehicles.

By experimental verification, we find that the initial center points in first partition and starting points for vehicles are close. Figure 5 and Table 3 show the experimental results on these two types of points. The red triangle points indicate the starting point of the two types of vehicles. The Cartesian coordinate (41,39) is the initial center point in the area. In the first place, it should be pointed out that the network density is changed after network partition. Now, we change the proportions of distance priority *α* and routing hop priority *β* in choosing starting position. Obviously, the starting position moves down when we decrease the proportion of routing hop. This is because the node density in left bottom is obviously higher than others parts. Thus, to reduce the distance consumption, the starting point must be move down. In addition, the starting position moves up when we increase the proportion of routing hop. This is because the nodes in top right corner are remote than others. Thus, to reduce routing hops influence of these nodes, the starting position should move up to maintain the routing hops minimally. Besides, we divide the network based on distance and routing hops with equal proportion. The node always chooses the initial center points with minimum distance and routing hops. Thus, when we change the proportion on distance and routing hop, the starting position will have corresponding changes. This shows that our algorithm of selecting starting position is very effective. Obviously, the starting point is almost at the center of each area, which effectively reduces the mobile energy consumption of the vehicles. Besides, we can verify that these nodes near starting position are likely to be chosen as anchors through anchor selection algorithm. This will reduce the forwarding data consumption greatly.

We study the convergence of the sub-algorithm according to the target function first. The iterative steps of Lagrange multipliers can ensure that data rate $r_{i}^{a}$ and link rate $f_{ij}^{a}$ converge to the optimal value. Then, we can get the amount of data producing by all nodes and data collected by DCV. Initially, it is assumed that the remaining battery energy of each node in the network is about 80–100% of the total energy and the battery energy of the nodes is randomly assigned. In the experiment, nine nodes are chosen as anchor points. For simplicity, we randomly select one of the anchor points and their nodes in the cluster as the reference object.

**Figure 6:**
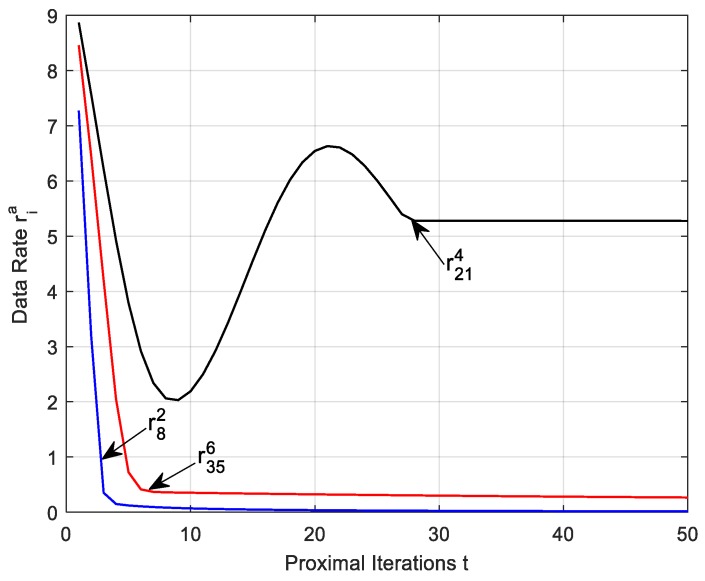
Convergence of data rates versus proximal *t*.

We first verify the data rate from node 21 to anchor 4, node 8 to anchor 2, and the node 35 to anchor 6 with a utility weight 1. Figure 6 shows the node data generation rate after several iterations. It can be seen from Figure 6 that data rates oscillate at the beginning, and then tend to fluctuate gently and achieve convergence finally. This is because the iteration step is larger in the initial stage, $r_{i}^{a}$ will be assigned to $y_{i}^{a}$ making $r_{i}^{a}$ change larger and the range of oscillation is more obvious after each iteration. With the increase of the number of iterations and reduce the iterative step size, the node data rate $r_{i}^{a}$ tends to be smooth, and the value of $y_{i}^{a}$ will go smoothly. Besides, the algorithm tends to achieve convergence and all variables go to convergence finally. This is an interactive process.

**Figure 7:**
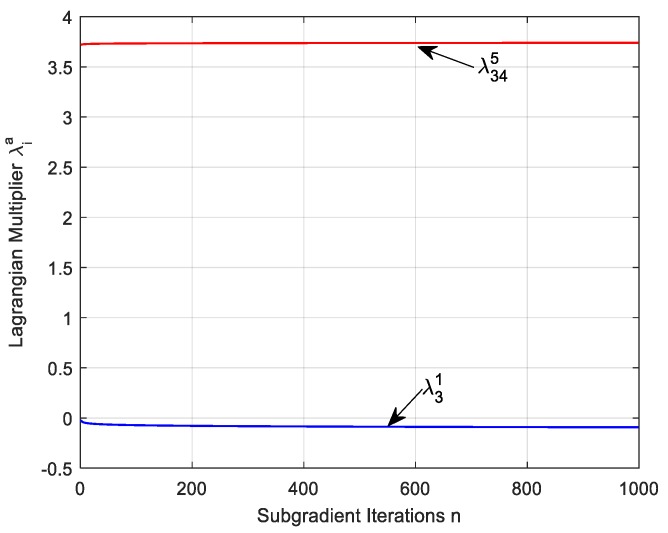
Convergence of Lagrange multiplier versus proximal n.

Figure 7 shows the evolution of Lagrange multiplier $\lambda_{i}^{a}$ versus the number of iterations. We verify the Lagrange multiplier from node 34 to anchor 5 and node 3 to anchor 1. We find that the lines can be seen as a straight line with less jitter. This is because although the data rate and adjacent link rate have a great impact on Lagrange multiplier, the multiplier has little change with each update iteration length smaller. In addition, in order to reduce the number of iterations of the calculation process, we can use the optimal Lagrange multiplier value of each node to compute directly data rate, link rate and the amount of data collected reducing the number of time complexity greatly.

### 5.2. Performance Analysis Based on Different Parameter Settings

**Figure 8:**
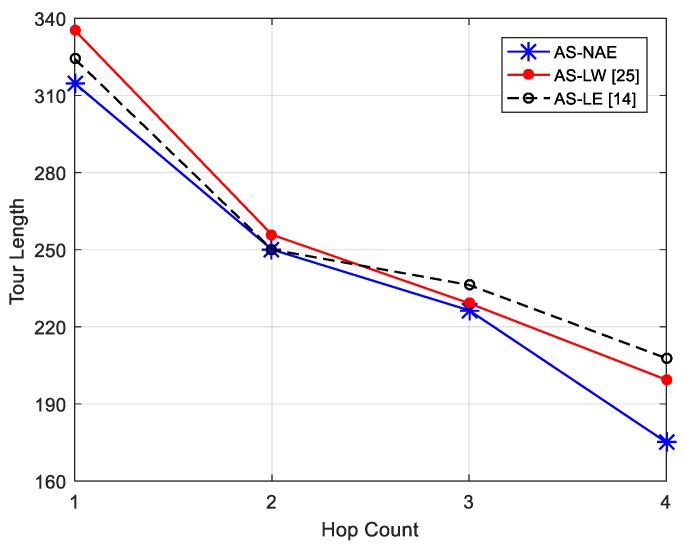
Impact of hop count on tour length.

To gain a better understanding of the impact of different parameter settings on the overall network performance, we first study the performance of different anchor selection algorithms as the function of cluster size *k* in terms tour of length. About 130 sensor nodes are uniformly distributed in the 85 m × 95 m sensing area after partitioning network and the sensing range and initial energy of each node remain unchanged in different anchor selection algorithm. We provide two schemes for performance comparison, including AS-LW and AS-LE. As mentioned earlier, in [25], the node with the largest weighted average was chosen as the anchor point, named AS-LW. In [15], authors chose the node with largest value as the anchor by constructing the least energy of each sensor’s neighbors within *k* hops, called AS-LE. As shown in Figure 8, obviously, the tour length of DCV decreases as the *k* increases. This is because the number of nodes within each anchors’ range increases as the cluster size increases, which decreases the number of anchor points and the tour length. Besides, we can see that the proposed algorithm can reduce the tour length effectively compared with the method of Based battery. This is because the Based battery method concentrates on the least battery energy within *k* hops. Thus, there is an extreme situation. The minimum battery energy within *k* hops will be same for multiple nodes closely. However, when $W_{i}$ and $W_{j}$ are equal for candidate anchor in anchor selection algorithm, where $\forall i,j\in n_{r}$, we choose the serial number of energy higher as anchor first to avoid the same scene in above methods. Thus, the proposed approach has an advantage gained over others.

**Figure 9:**
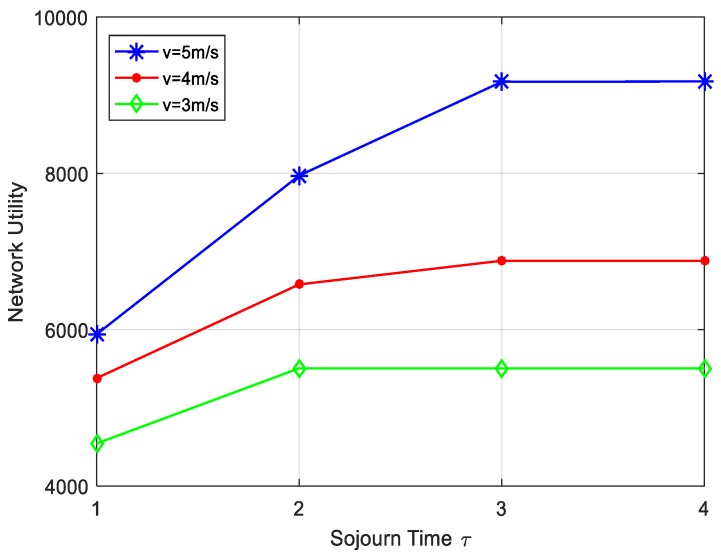
Impact of sojourn time and vehicle speed on network utility.

**Figure 10:**
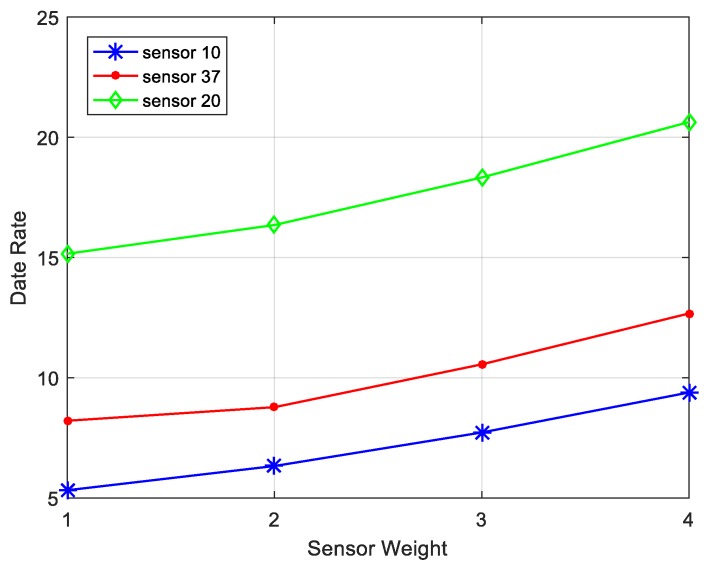
Impact of node weight on data rate.

In this paper, network utility refers to the amount of data generated by all nodes in the sensing area. There are a series of factors that could have an important effect on network performance, for example, utility weight, DCV speed and the sojourn time at each site. Figure 9 shows the impact on the network performance while movement speed of the DCV and the sojourn time at each site changing. Assume that the data collection cycle is dynamic, which means DCV tours the entire network as a collection cycle. It is clear that data amount is more while keeping the speed of DCV unchanged and sojourn time increasing. By adjusting the sojourn time at each site, DCV can collect more data until all data collected meaning the line smoothly. Besides, we consider the effect of different moving speeds on the overall network performance when the sojourn at each anchor is same. In a fixed collection cycle, the DCV can tour many times in overall network. Thus, more data can be collected as the speed of DCV increases. Figure 10 shows the impact of utility weight on data rate. Obviously, to keep the weights of other nodes unchanged, we find that the data rate increases with higher utility weight keeping the utility weight of other nodes unchanged. In addition, we can use the nodes with higher weights as anchor points to avoid the energy consumption on sending and receiving data and reduce the delay of data collection in the process of data collection.

## 6. Conclusions and Future Work

In this paper, we have studied joint design data collection and wireless charging by using mobile vehicles in each area. We first proposed a twice-partition algorithm based on center points, which could divide the network into several parts to avoid problematic schedules or stranded vehicles. It also simplified the calculation complexity. Then, we developed an algorithm based on distance and routing hops to find a starting position for DCV and WCV in each area. It could efficiently reduce the moving and forwarding data energy consumption by experimental results. In addition, AS-NAE was introduced to achieve a desirable balance between data amount and data latency. We formulated data collection problem into an optimization problem in which DCV sojourns a constant time at each anchor to collect data. Then, each sensor tuned the data rate and link rate based on energy status to maximize data amounts. Because each node has different energy consumption rate according to different data packet amounts on forwarding or receiving, we proposed an adaptive energy threshold to maintain network running continuously. Finally, we provided extensive numerical results to prove the effectiveness of the proposed algorithm.

To combine the wireless charging and mobile data collection better, some issues need to be settled in the future. In this paper, we assume that the flow rates are dynamic without consideration of link choice problem. This causes some flow rates to be empty, thus no data packets are transmitted, which will lead to many waste links. Thus, we will consider how to schedule link efficiently avoiding empty packets transmission case in some link. Second, in this paper, the vehicle cannot communicate with two or more nodes simultaneously. Thus, it is difficult to arrange the nodes pair, antenna of vehicles, tour path of vehicles to increase charging efficiency and data collection amount through anchors. We plan to study these problems in the future.

## Figures and Tables

**Table 1:** List of notations.

Notation	Definition
*N*	Set of sensor nodes in the whole network
*n_r_*	Set of sensor nodes in a cell after network partition
*A*	Set of anchors points in a cell
$r_{i}^{a}$	Data rate of sensor *i* when DCV is at anchor point *a*
$f_{ij}^{a}$	Link rate over (*i*, *j*) when DCV is at anchor point *a*
*P_i,a_*	Set of parent nodes of sensor *i* for anchor *a*
*C_i,a_*	Set of children nodes of sensor *i* for anchor *a*
*τ*_a_	Sojourn time of DCV at anchor *a* in a migration tour
*L_b_*	Upper bound of migration tour
*L_tsp_*	Tour length in a migration tour
*T*	Data collection cycle
*C_b_*	Residual energy of node
*C_v_*	Residual energy of vehicle
*C_r_*	Max energy of each vehicle
*C_s_*	Max energy of node
*e_t_*, *e_r_*	Energy consumed for transmitting or receiving a unit flow
*e_s_*	Energy consumed for generating and sensing a unit flow
*v*	Moving velocity of the vehicle

**Table 2:** Parameter settings.

Parameter	Value
*C_r_*	10 J
*C_s_*	20 KJ
*e_s_*	0.05 mJ
*e_r_,e_t_*	0.3 mJ
*v*	3–5 m/s
*π*	10 Kbits

**Table 3:** Proportions of distance priority *α* and routing hop priority *β*.

α, β	Coordinate	α, β	Coordinate
0.5,0.5	(41,39)	0.5,0.5	(41,39)
0.6,0.4	(34,35)	0.4,0.6	(41,39)
0.7,0.3	(34,35)	0.3,0.7	(41,39)
0.8,0.2	(34,35)	0.2,0.8	(41,39)
0.9,0.1	(40,32)	0.1,0.9	(33,44)
1,0	(40,32)	0,1	(33,44)
